# Diaquatetra­kis(μ-3-meth­oxy­benzoato-κ^2^ *O* ^1^:*O* ^1′^)dicopper(II)

**DOI:** 10.1107/S2414314620004484

**Published:** 2020-04-07

**Authors:** Bikshandarkoil R. Srinivasan, Pooja H. Bhargao, P. K. Sudhadevi

**Affiliations:** a School of Chemical Sciences, Goa University, Goa 403206, India; b Sophisticated Analytical Instruments Facility (SAIF), Indian Institute of Technology Madras, Chennai 600036, India; Vienna University of Technology, Austria

**Keywords:** crystal structure, paddle-wheel structure, binuclear copper complex, hydrogen bonding

## Abstract

The asymmetric unit of the binuclear title compound, [Cu_2_(C_8_H_7_O_3_)_4_(H_2_O)_2_], comprises two halves of diaquatetra­kis­(μ-3-meth­oxy­benzoato-κ^2^ *O* ^1^:*O* ^1′^)dicopper(II) units. The paddle-wheel structure of each complex is completed by application of inversion symmetry, with the inversion centre situated at the midpoint between two Cu^II^ atoms in each dimer. The two Cu^II^ atoms of each centrosymmetric dimer are bridged by four 3-meth­oxy­benzoate anions resulting in Cu⋯Cu separations of 2.5961 (11) and 2.6060 (12) Å, respectively. The square-pyramidal coordination sphere of each Cu^II^ atom is completed by an apical water mol­ecule. Inter­molecular O—H⋯O hydrogen bonds of weak nature link the complexes into layers parallel to (100). The three-dimensional network structure is accomplished by C—H⋯O hydrogen bonds inter­linking adjacent layers.

## Abstract 2

In the crystal structure of the title compound two independent binuclear copper(II) complexes are present, each with site symmetry 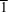 and with the Cu^II^ atoms in a square-pyramidal coordination environment.

## Graphical abstract

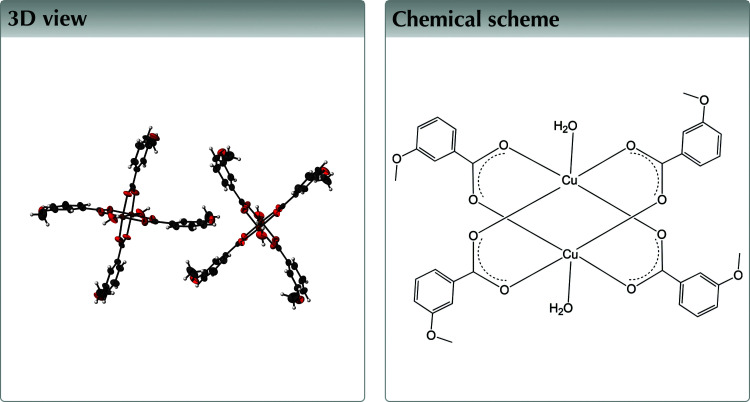

## Structure description

A very early structure investigation of cupric acetate monohydrate revealed that it is dimeric in nature, represented by the formula [Cu_2_(CH_3_COO)_4_(H_2_O)_2_] (Van Niekerk & Schoening, 1953 ▸). In the dimer, each of the two cupric ions is bonded to four oxygen atoms of four bridging acetate ligands in addition to a terminal aqua ligand. This kind of coordination, wherein a pair of metal cations is bonded to four symmetrically bridging carboxyl­ate anions, is referred to as a paddle-wheel structure and is well documented for several dimeric copper carboxyl­ates (Doedens, 1976 ▸). The Cambridge Structural Database (CSD, version 5.40, update September 2019; Groom *et al.*, 2016 ▸) lists the structures of several dicopper(II) compounds where the cupric cations are symmetrically bridged by four carboxyl­ate ligands. The fifth ligand can be a terminal water mol­ecule or any O– or N-donor ligand. A dinuclear copper compound with a paddle-wheel structure, *viz*. tetra­kis­(μ-3-meth­oxy­benzoato- κ^2^ *O* ^1^:*O* ^1′^)bis­[aceto­nitrile­copper(II)] (**2**), was reported previously for the meth­oxy­benzoate anion (Kar *et al.*, 2011 ▸). In the present study, we describe the structure of a related dinuclear copper complex where the aceto­nitrile ligands are replaced by aqua ligands.

**Figure 1:**
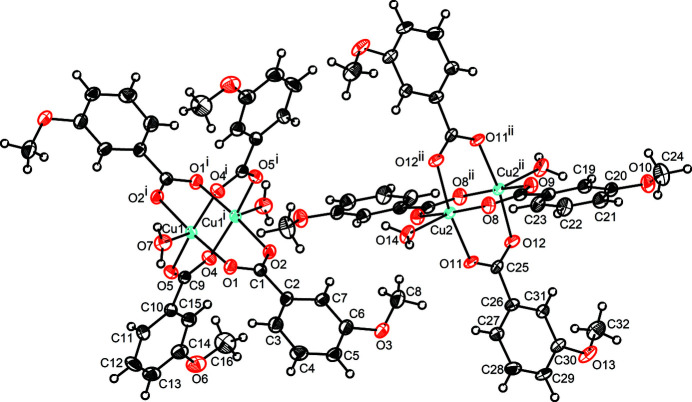
The two centrosymmetric binuclear complexes in the crystal structure of [Cu_2_(C_8_H_7_O_3_)_4_(H_2_O)_2_] with displacement ellipsoids drawn at the 30% probability level. [Symmetry codes: (i) −*x* + 1, −*y* + 1, −*z*; (ii) −*x* + 2, −*y* + 1, −*z* + 1.]

The crystal structure of the title compound, [Cu_2_(C_8_H_7_O_3_)_4_(H_2_O)_2_], (**1**), consists of two crystallographically unique cupric cations, four crystallographically independent 3-meth­oxy­benzoate anions and two terminal water mol­ecules that build up two independent halves of a dimeric [Cu_2_(C_8_H_7_O_3_)_4_(H_2_O)_2_] complex, the other halves being generated by inversion symmetry. The inversion centre is situated at the midpoint of the line connecting two Cu^II^ atoms in each of the dimers (Fig. 1 ▸). In each centrosymmetric dimer, a pair of Cu^II^ atoms is connected through four *syn*–*syn* bis-monodentate 3-meth­oxy­benzoate bridges to generate a binuclear paddle-wheel unit. The fifth ligand, O7 on Cu1 and O14 on Cu2, is a terminal water mol­ecule, defining an overall square-pyramidal coordination sphere around the central metal cation. Bond lengths and angles of the 3-meth­oxy­benzoate anions are in normal ranges and are in agreement with reported data (Kar *et al.*, 2011 ▸). The Cu—O_water_ bonds [2.171 (4) and 2.126 (4) Å for Cu1 and Cu2, respectively] are elongated as compared to the Cu—O_carboxyl­ate_ distances ranging from 1.949 (4) to 1.959 (3) Å for Cu1 and from 1.936 (3) to 1.973 (3) Å for Cu2. The Cu⋯Cu separations in the dimers amount to 2.6060 (12) Å for Cu1 and 2.5961 (11) Å for Cu2, which are shorter than the Cu⋯Cu distance of 2.6433 (3) Å reported for (**2**) (Kar *et al.*, 2011 ▸).

**Figure 2:**
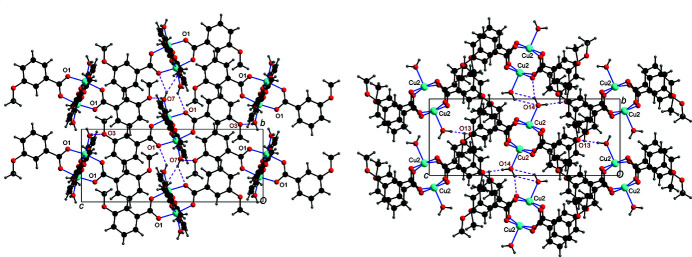
A view along [100] showing the O—H⋯O hydrogen bonds (dashed lines) around the Cu1 dimer (left) and the Cu2 dimer (right).

**Figure 3:**
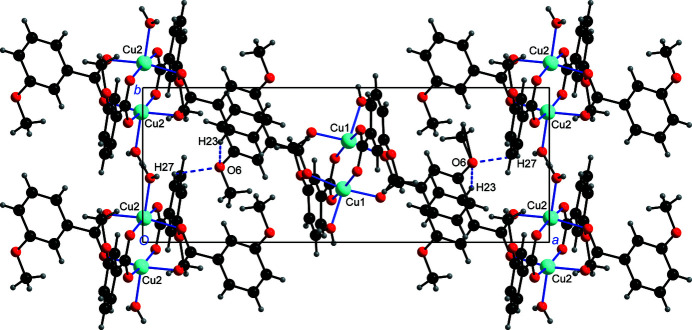
A view along [001] showing the inter­linking of dimeric Cu1 units with adjacent dimeric Cu2 units with the aid of C—H⋯O hydrogen bonds.

The water mol­ecules, and the phenyl groups C23—H23 and C27—H27, respectively, function as hydrogen-bond donors, while the meth­oxy oxygen atoms O3, O6 and O13 and the carboxyl­ate oxygen atoms O1, O5, O11 and O14 function as hydrogen-bond acceptors; parts of the O—H⋯O hydrogen bonds are bifurcated (Table 1 ▸). Each Cu1 dimer is linked to six other symmetry-related Cu1 dimers with the aid of three O—H⋯O hydrogen bonds, and each Cu2 dimer is hydrogen-bonded to six other symmetry-related Cu2 dimers (Fig. 2 ▸). As a result, O—H⋯O hydrogen-bonded layers parallel to (100) are formed. The two C—H⋯O hydrogen bonds inter­link adjacent layers into a three-dimensional network (Fig. 3 ▸).

## Synthesis and crystallization

Cupric oxide (100 mg) was added in small portions to a hot aqueous solution of 3-meth­oxy­benzoic acid (0.304 g, 2 mmol) in water (100 ml). The hot reaction mixture was continuously stirred to dissolve the oxide. When most of the oxide had dissolved, the blue reaction mixture was filtered to remove the insoluble matter. The blue filtrate thus obtained was left aside for crystallization. After a few days blue–greenish crystals of (**1**) slowly separated. The crystals were filtered and dried in air. Yield 35%.

## Refinement

Crystal data, data collection and structure refinement details are summarized in Table 2 ▸. The crystal under investigation was a two-component twin with a refined batch scale factor (BASF) of 0.47. The matrix that was used for overlapping the twin domains is (101 0 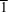 0 00 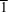 ). H atoms of water mol­ecules were discernible from a difference-Fourier map. To get a reasonable shape, water mol­ecules were refined with a target value of 0.85 (2) Å for O—H bond lengths and of 1.35 (2) Å for H⋯H distances.

## Supplementary Material

Crystal structure: contains datablock(s) I, global. DOI: 10.1107/S2414314620004484/wm4126sup1.cif

Structure factors: contains datablock(s) I. DOI: 10.1107/S2414314620004484/wm4126Isup3.hkl

CCDC reference: 1993956

Additional supporting information:  crystallographic information; 3D view; checkCIF report

## Figures and Tables

**Table 1:** Hydrogen-bond geometry (Å, °)

*D*—H⋯*A*	*D*—H	H⋯*A*	*D*⋯*A*	*D*—H⋯*A*
O7—H7*A*⋯O3^i^	0.84 (2)	2.11 (3)	2.912 (6)	161 (5)
O7—H7*B*⋯O1^ii^	0.83 (2)	2.42 (5)	3.107 (6)	141 (7)
O7—H7*B*⋯O5^ii^	0.83 (2)	2.60 (5)	3.306 (6)	144 (6)
O14—H14*B*⋯O13^iii^	0.84 (2)	2.00 (2)	2.831 (6)	169 (8)
O14—H14*A*⋯O11^iv^	0.83 (2)	2.11 (3)	2.905 (5)	160 (6)
O14—H14*A*⋯O14^iv^	0.83 (2)	2.57 (5)	3.055 (8)	118 (5)
C23—H23⋯O6^v^	0.93	2.52	3.355 (7)	149
C27—H27⋯O6^vi^	0.93	2.57	3.114 (7)	117

Symmetry codes: (i) 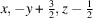 ; (ii) 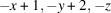 ; (iii) 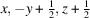 ; (iv) 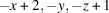 ; (v) 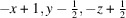 ; (vi) 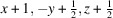 .

**Table 2:** Experimental details

Crystal data	
Chemical formula	[Cu_2_(C_8_H_7_O_3_)_4_(H_2_O)_2_]
*M*_r_	767.65
Crystal system, space group	Monoclinic, *P*2_1_/*c*
Temperature (K)	296
*a*, *b*, *c* (Å)	22.515 (3), 7.5349 (6), 21.536 (2)
β (°)	118.429 (4)
*V* (Å^3^)	3213.0 (6)
*Z*	4
Radiation type	Mo *K*α
μ (mm^−1^)	1.40
Crystal size (mm)	0.20 × 0.20 × 0.15

Data collection	
Diffractometer	Bruker AXS Kappa APEXII CCD
Absorption correction	Multi-scan (*SADABS*; Bruker, 2012 ▸)
*T*_min_, *T*_max_	0.410, 0.745
No. of measured, independent and observed [*I* > 2σ(*I*)] reflections	36309, 6704, 5194
*R*_int_	0.082
(sin θ/λ)_max_ (Å^−1^)	0.631

Refinement	
*R*[*F* ^2^ > 2σ(*F* ^2^)], *wR*(*F* ^2^), *S*	0.043, 0.108, 1.02
No. of reflections	6704
No. of parameters	454
No. of restraints	6
H-atom treatment	H atoms treated by a mixture of independent and constrained refinement
Δρ_max_, Δρ_min_ (e Å^−3^)	0.90, −0.83

Computer programs: *APEX2* and *SAINT* (Bruker, 2012 ▸), *SHELXT* (Sheldrick, 2015*a* ▸), *SHELXL* (Sheldrick, 2015*b* ▸), *DIAMOND* (Brandenburg, 1999 ▸) and *publCIF* (Westrip, 2010 ▸).

## full crystallographic data

### Crystal data

**Table:**

[Cu_2_(C_8_H_7_O_3_)_4_(H_2_O)_2_]	*F*(000) = 1576
*M**_r_* = 767.65	*D*_x_ = 1.587 Mg m^−^^3^
Monoclinic, *P*2_1_/*c*	Mo *K*α radiation, λ = 0.71073 Å
*a* = 22.515 (3) Å	Cell parameters from 9989 reflections
*b* = 7.5349 (6) Å	θ = 2.2–26.2°
*c* = 21.536 (2) Å	µ = 1.40 mm^−^^1^
β = 118.429 (4)°	*T* = 296 K
*V* = 3213.0 (6) Å^3^	Block, bluish green
*Z* = 4	0.20 × 0.20 × 0.15 mm

### Data collection

**Table:**

Bruker AXS Kappa APEXII CCD diffractometer	6704 independent reflections
Radiation source: fine-focus sealed tube	5194 reflections with *I* > 2σ(*I*)
Graphite monochromator	*R*_int_ = 0.082
ω and φ scan	θ_max_ = 26.6°, θ_min_ = 1.0°
Absorption correction: multi-scan (*SADABS*; Bruker, 2012)	*h* = −28→28
*T*_min_ = 0.410, *T*_max_ = 0.745	*k* = −9→9
36309 measured reflections	*l* = −26→26

### Refinement

**Table:**

Refinement on *F*^2^	6 restraints
Least-squares matrix: full	Hydrogen site location: mixed
*R*[*F*^2^ > 2σ(*F*^2^)] = 0.043	H atoms treated by a mixture of independent and constrained refinement
*wR*(*F*^2^) = 0.108	*w* = 1/[σ^2^(*F*_o_^2^) + (0.0351*P*)^2^ + 3.0856*P*] where *P* = (*F*_o_^2^ + 2*F*_c_^2^)/3
*S* = 1.02	(Δ/σ)_max_ = 0.002
6704 reflections	Δρ_max_ = 0.90 e Å^−^^3^
454 parameters	Δρ_min_ = −0.83 e Å^−^^3^

### Special details

**Table:**

Geometry. All esds (except the esd in the dihedral angle between two l.s. planes) are estimated using the full covariance matrix. The cell esds are taken into account individually in the estimation of esds in distances, angles and torsion angles; correlations between esds in cell parameters are only used when they are defined by crystal symmetry. An approximate (isotropic) treatment of cell esds is used for estimating esds involving l.s. planes.
Refinement. Refined as a two-component twin

### Fractional atomic coordinates and isotropic or equivalent isotropic displacement parameters (Å^2^)

**Table:**

	*x*	*y*	*z*	*U*_iso_*/*U*_eq_	
Cu1	0.50684 (3)	0.65510 (8)	−0.02260 (3)	0.03022 (19)	
C1	0.5402 (2)	0.6223 (7)	0.1219 (3)	0.0341 (12)	
C2	0.5610 (2)	0.7024 (8)	0.1931 (3)	0.0352 (12)	
C3	0.5619 (3)	0.8829 (7)	0.2013 (3)	0.0473 (14)	
H3	0.553854	0.957326	0.163685	0.057*	
C4	0.5745 (3)	0.9535 (8)	0.2649 (3)	0.0565 (16)	
H4	0.572505	1.075719	0.269696	0.068*	
C5	0.5900 (3)	0.8450 (8)	0.3216 (3)	0.0483 (15)	
H5	0.598563	0.893345	0.364783	0.058*	
C6	0.5929 (3)	0.6635 (7)	0.3145 (3)	0.0451 (14)	
C7	0.5772 (3)	0.5917 (8)	0.2496 (3)	0.0409 (13)	
H7	0.577578	0.469341	0.244079	0.049*	
C8	0.6299 (5)	0.3893 (9)	0.3737 (4)	0.095 (3)	
H8A	0.594361	0.328194	0.334527	0.142*	
H8B	0.637957	0.332541	0.416971	0.142*	
H8C	0.670353	0.385973	0.369359	0.142*	
C9	0.3827 (2)	0.5945 (7)	−0.0264 (3)	0.0370 (11)	
C10	0.3150 (3)	0.6464 (7)	−0.0354 (3)	0.0381 (12)	
C11	0.2869 (3)	0.8092 (8)	−0.0627 (3)	0.0465 (14)	
H11	0.308308	0.886444	−0.079308	0.056*	
C12	0.2266 (3)	0.8568 (8)	−0.0650 (3)	0.0604 (17)	
H12	0.206701	0.964655	−0.085164	0.073*	
C13	0.1958 (3)	0.7476 (9)	−0.0382 (4)	0.0584 (19)	
H13	0.155816	0.782540	−0.038909	0.070*	
C14	0.2244 (3)	0.5856 (9)	−0.0100 (3)	0.0526 (15)	
C15	0.2829 (3)	0.5331 (8)	−0.0094 (3)	0.0457 (13)	
H15	0.301205	0.422211	0.008402	0.055*	
C16	0.2158 (4)	0.3189 (9)	0.0455 (5)	0.083 (2)	
H16A	0.211575	0.238074	0.009184	0.124*	
H16B	0.191200	0.273485	0.068286	0.124*	
H16C	0.262557	0.331491	0.079654	0.124*	
O1	0.53588 (19)	0.7269 (5)	0.0746 (2)	0.0403 (10)	
O2	0.52749 (18)	0.4584 (4)	0.11476 (17)	0.0380 (8)	
O3	0.6108 (2)	0.5671 (5)	0.3743 (2)	0.0639 (12)	
O4	0.40388 (17)	0.4427 (5)	−0.00258 (19)	0.0419 (9)	
O5	0.41505 (18)	0.7099 (5)	−0.04147 (19)	0.0412 (8)	
O6	0.1892 (2)	0.4862 (6)	0.0153 (2)	0.0651 (12)	
O7	0.5378 (3)	0.9096 (5)	−0.0449 (3)	0.0582 (11)	
H7A	0.566 (2)	0.927 (7)	−0.059 (3)	0.050 (18)*	
H7B	0.530 (3)	1.005 (5)	−0.031 (4)	0.10 (3)*	
Cu2	1.00357 (3)	0.34185 (7)	0.52545 (3)	0.02704 (17)	
C17	0.8821 (2)	0.4364 (7)	0.4052 (3)	0.0344 (11)	
C18	0.8143 (2)	0.3902 (6)	0.3454 (3)	0.0335 (11)	
C19	0.7823 (2)	0.5098 (7)	0.2908 (3)	0.0378 (11)	
H19	0.800444	0.622163	0.293246	0.045*	
C20	0.7232 (3)	0.4598 (7)	0.2326 (3)	0.0416 (12)	
C21	0.6958 (3)	0.2923 (9)	0.2304 (3)	0.0511 (15)	
H21	0.655720	0.258591	0.191294	0.061*	
C22	0.7275 (3)	0.1787 (7)	0.2854 (3)	0.0509 (14)	
H22	0.708932	0.067457	0.283747	0.061*	
C23	0.7873 (3)	0.2266 (8)	0.3437 (3)	0.0450 (15)	
H23	0.808799	0.148658	0.381440	0.054*	
C24	0.7133 (3)	0.7314 (10)	0.1710 (4)	0.073 (2)	
H24A	0.721094	0.799866	0.211808	0.110*	
H24B	0.680916	0.791342	0.129157	0.110*	
H24C	0.754901	0.717887	0.169388	0.110*	
C25	1.0434 (2)	0.3824 (7)	0.4183 (2)	0.0327 (11)	
C26	1.0650 (2)	0.3142 (7)	0.3676 (3)	0.0334 (11)	
C27	1.0786 (3)	0.1352 (7)	0.3666 (3)	0.0438 (13)	
H27	1.077861	0.058767	0.400195	0.053*	
C28	1.0931 (3)	0.0720 (8)	0.3153 (3)	0.0532 (16)	
H28	1.101825	−0.048261	0.314230	0.064*	
C29	1.0948 (3)	0.1821 (8)	0.2661 (3)	0.0499 (15)	
H29	1.103129	0.136388	0.230861	0.060*	
C30	1.0841 (3)	0.3626 (7)	0.2686 (3)	0.0400 (13)	
C31	1.0693 (2)	0.4274 (7)	0.3186 (3)	0.0342 (12)	
H31	1.061863	0.548415	0.320089	0.041*	
C32	1.1014 (4)	0.6486 (8)	0.2328 (4)	0.0647 (18)	
H32A	1.063753	0.700448	0.235440	0.097*	
H32B	1.106403	0.703643	0.195349	0.097*	
H32C	1.141820	0.666487	0.276769	0.097*	
O8	0.91180 (17)	0.3186 (4)	0.44982 (19)	0.0428 (9)	
O9	0.90509 (18)	0.5881 (4)	0.40517 (17)	0.0416 (9)	
O10	0.68798 (18)	0.5618 (5)	0.1750 (2)	0.0544 (10)	
O11	1.0361 (2)	0.2734 (4)	0.4584 (2)	0.0385 (9)	
O12	1.0316 (2)	0.5454 (4)	0.41646 (19)	0.0408 (9)	
O13	1.0901 (2)	0.4638 (5)	0.2193 (2)	0.0610 (12)	
O14	1.0181 (3)	0.0849 (5)	0.5712 (2)	0.0567 (12)	
H14B	1.034 (4)	0.074 (8)	0.6150 (11)	0.10 (3)*	
H14A	0.998 (3)	−0.009 (6)	0.552 (3)	0.09 (2)*	

### Atomic displacement parameters (Å^2^)

**Table:**

	*U*^11^	*U*^22^	*U*^33^	*U*^12^	*U*^13^	*U*^23^
Cu1	0.0338 (4)	0.0263 (3)	0.0275 (4)	0.0029 (2)	0.0122 (3)	0.0011 (2)
C1	0.019 (2)	0.050 (3)	0.029 (3)	0.004 (2)	0.008 (2)	−0.002 (2)
C2	0.025 (3)	0.050 (3)	0.027 (3)	−0.004 (2)	0.010 (2)	−0.001 (2)
C3	0.051 (3)	0.044 (3)	0.043 (3)	−0.001 (3)	0.019 (3)	0.000 (3)
C4	0.066 (4)	0.043 (3)	0.059 (4)	−0.001 (3)	0.029 (3)	−0.017 (3)
C5	0.045 (3)	0.061 (4)	0.034 (3)	0.001 (3)	0.014 (3)	−0.016 (3)
C6	0.041 (3)	0.058 (3)	0.035 (3)	0.001 (3)	0.017 (3)	0.000 (3)
C7	0.045 (3)	0.047 (3)	0.033 (3)	0.000 (2)	0.020 (3)	−0.007 (2)
C8	0.157 (8)	0.080 (5)	0.048 (4)	0.050 (5)	0.050 (5)	0.021 (4)
C9	0.032 (3)	0.049 (3)	0.025 (2)	0.007 (2)	0.009 (2)	−0.004 (2)
C10	0.032 (3)	0.045 (3)	0.029 (3)	0.006 (2)	0.008 (2)	−0.005 (2)
C11	0.038 (3)	0.056 (4)	0.038 (3)	0.011 (3)	0.012 (2)	0.004 (3)
C12	0.040 (3)	0.068 (4)	0.060 (4)	0.025 (3)	0.013 (3)	−0.003 (3)
C13	0.033 (4)	0.086 (5)	0.056 (4)	0.008 (3)	0.020 (3)	−0.016 (3)
C14	0.034 (3)	0.075 (4)	0.048 (3)	0.000 (3)	0.018 (3)	−0.014 (3)
C15	0.039 (3)	0.055 (3)	0.036 (3)	0.001 (3)	0.012 (2)	−0.010 (3)
C16	0.080 (5)	0.078 (5)	0.103 (7)	−0.007 (4)	0.053 (5)	0.010 (5)
O1	0.047 (3)	0.0385 (19)	0.030 (2)	−0.0018 (16)	0.0142 (19)	−0.0012 (16)
O2	0.044 (2)	0.0358 (19)	0.0291 (19)	−0.0007 (16)	0.0129 (16)	−0.0034 (15)
O3	0.095 (3)	0.066 (3)	0.035 (2)	0.022 (2)	0.034 (2)	0.004 (2)
O4	0.0324 (18)	0.044 (2)	0.046 (2)	0.0060 (16)	0.0161 (16)	0.0035 (17)
O5	0.040 (2)	0.042 (2)	0.045 (2)	0.0090 (17)	0.0218 (18)	0.0052 (17)
O6	0.047 (2)	0.082 (3)	0.072 (3)	−0.008 (2)	0.033 (2)	−0.015 (3)
O7	0.092 (4)	0.031 (2)	0.070 (3)	0.001 (2)	0.053 (3)	0.009 (2)
Cu2	0.0363 (4)	0.0242 (3)	0.0232 (3)	−0.0029 (2)	0.0163 (3)	−0.0015 (2)
C17	0.033 (3)	0.044 (3)	0.030 (3)	0.000 (2)	0.018 (2)	−0.005 (2)
C18	0.031 (2)	0.040 (3)	0.033 (3)	−0.005 (2)	0.018 (2)	−0.005 (2)
C19	0.033 (3)	0.044 (3)	0.034 (3)	−0.005 (2)	0.014 (2)	−0.007 (2)
C20	0.033 (3)	0.058 (3)	0.030 (3)	0.004 (2)	0.012 (2)	−0.001 (2)
C21	0.042 (4)	0.062 (4)	0.041 (4)	−0.008 (3)	0.014 (3)	−0.010 (3)
C22	0.045 (3)	0.053 (3)	0.046 (3)	−0.019 (3)	0.015 (3)	−0.005 (3)
C23	0.045 (4)	0.051 (3)	0.034 (3)	−0.009 (3)	0.016 (3)	−0.005 (3)
C24	0.065 (4)	0.065 (4)	0.070 (5)	−0.001 (4)	0.016 (4)	0.018 (4)
C25	0.036 (3)	0.041 (3)	0.021 (2)	−0.005 (2)	0.014 (2)	−0.004 (2)
C26	0.032 (3)	0.041 (3)	0.033 (3)	−0.007 (2)	0.019 (2)	−0.009 (2)
C27	0.054 (3)	0.043 (3)	0.038 (3)	0.005 (3)	0.025 (3)	−0.001 (2)
C28	0.074 (4)	0.042 (3)	0.054 (4)	0.011 (3)	0.039 (3)	−0.004 (3)
C29	0.063 (4)	0.059 (4)	0.042 (3)	0.004 (3)	0.037 (3)	−0.011 (3)
C30	0.045 (3)	0.049 (3)	0.032 (3)	−0.010 (3)	0.023 (3)	−0.010 (2)
C31	0.039 (3)	0.035 (3)	0.029 (3)	−0.004 (2)	0.016 (2)	−0.002 (2)
C32	0.084 (5)	0.062 (4)	0.060 (4)	−0.022 (3)	0.044 (4)	−0.005 (3)
O8	0.038 (2)	0.044 (2)	0.038 (2)	−0.0101 (17)	0.0114 (17)	−0.0004 (17)
O9	0.041 (2)	0.0380 (18)	0.0358 (19)	−0.0083 (16)	0.0105 (17)	−0.0045 (16)
O10	0.044 (2)	0.059 (2)	0.042 (2)	0.0012 (18)	0.0057 (18)	0.0023 (19)
O11	0.060 (3)	0.0358 (18)	0.033 (2)	−0.0010 (17)	0.033 (2)	−0.0042 (15)
O12	0.065 (3)	0.0319 (19)	0.040 (2)	−0.0024 (17)	0.036 (2)	−0.0039 (15)
O13	0.099 (3)	0.058 (2)	0.047 (2)	−0.019 (2)	0.051 (2)	−0.013 (2)
O14	0.106 (4)	0.0252 (19)	0.036 (2)	−0.012 (2)	0.032 (2)	−0.0034 (17)

### Geometric parameters (Å, º)

**Table:**

Cu1—O1	1.949 (4)	Cu2—O8	1.936 (3)
Cu1—O2^i^	1.951 (3)	Cu2—O9^ii^	1.953 (3)
Cu1—O5	1.951 (3)	Cu2—O12^ii^	1.964 (3)
Cu1—O4^i^	1.959 (3)	Cu2—O11	1.973 (3)
Cu1—O7	2.171 (4)	Cu2—O14	2.126 (4)
Cu1—Cu1^i^	2.6060 (12)	Cu2—Cu2^ii^	2.5961 (11)
C1—O1	1.254 (6)	C17—O8	1.244 (6)
C1—O2	1.261 (6)	C17—O9	1.255 (6)
C1—C2	1.500 (7)	C17—C18	1.496 (7)
C2—C3	1.370 (7)	C18—C23	1.367 (7)
C2—C7	1.374 (8)	C18—C19	1.381 (7)
C3—C4	1.367 (9)	C19—C20	1.377 (7)
C3—H3	0.9300	C19—H19	0.9300
C4—C5	1.369 (8)	C20—O10	1.351 (6)
C4—H4	0.9300	C20—C21	1.396 (8)
C5—C6	1.381 (7)	C21—C22	1.355 (8)
C5—H5	0.9300	C21—H21	0.9300
C6—O3	1.362 (7)	C22—C23	1.382 (8)
C6—C7	1.378 (8)	C22—H22	0.9300
C7—H7	0.9300	C23—H23	0.9300
C8—O3	1.409 (7)	C24—O10	1.418 (8)
C8—H8A	0.9600	C24—H24A	0.9600
C8—H8B	0.9600	C24—H24B	0.9600
C8—H8C	0.9600	C24—H24C	0.9600
C9—O4	1.252 (6)	C25—O12	1.253 (6)
C9—O5	1.272 (6)	C25—O11	1.258 (6)
C9—C10	1.495 (7)	C25—C26	1.484 (7)
C10—C11	1.377 (7)	C26—C27	1.385 (7)
C10—C15	1.397 (7)	C26—C31	1.395 (7)
C11—C12	1.380 (8)	C27—C28	1.379 (8)
C11—H11	0.9300	C27—H27	0.9300
C12—C13	1.370 (9)	C28—C29	1.361 (8)
C12—H12	0.9300	C28—H28	0.9300
C13—C14	1.379 (9)	C29—C30	1.387 (8)
C13—H13	0.9300	C29—H29	0.9300
C14—C15	1.369 (8)	C30—C31	1.361 (7)
C14—O6	1.376 (7)	C30—O13	1.365 (7)
C15—H15	0.9300	C31—H31	0.9300
C16—O6	1.414 (8)	C32—O13	1.420 (6)
C16—H16A	0.9600	C32—H32A	0.9600
C16—H16B	0.9600	C32—H32B	0.9600
C16—H16C	0.9600	C32—H32C	0.9600
O7—H7A	0.835 (19)	O14—H14B	0.84 (2)
O7—H7B	0.830 (19)	O14—H14A	0.832 (19)
			
O1—Cu1—O2^i^	169.12 (15)	O8—Cu2—O9^ii^	168.91 (14)
O1—Cu1—O5	86.87 (16)	O8—Cu2—O12^ii^	89.02 (16)
O2^i^—Cu1—O5	90.77 (15)	O9^ii^—Cu2—O12^ii^	89.57 (16)
O1—Cu1—O4^i^	91.82 (16)	O8—Cu2—O11	88.84 (16)
O2^i^—Cu1—O4^i^	88.52 (15)	O9^ii^—Cu2—O11	90.47 (16)
O5—Cu1—O4^i^	169.26 (15)	O12^ii^—Cu2—O11	169.05 (14)
O1—Cu1—O7	90.79 (17)	O8—Cu2—O14	100.04 (17)
O2^i^—Cu1—O7	100.09 (17)	O9^ii^—Cu2—O14	91.05 (17)
O5—Cu1—O7	100.79 (17)	O12^ii^—Cu2—O14	96.79 (16)
O4^i^—Cu1—O7	89.89 (17)	O11—Cu2—O14	94.16 (15)
O1—Cu1—Cu1^i^	83.50 (11)	O8—Cu2—Cu2^ii^	84.33 (10)
O2^i^—Cu1—Cu1^i^	85.81 (10)	O9^ii^—Cu2—Cu2^ii^	84.59 (10)
O5—Cu1—Cu1^i^	88.03 (11)	O12^ii^—Cu2—Cu2^ii^	84.78 (10)
O4^i^—Cu1—Cu1^i^	81.23 (11)	O11—Cu2—Cu2^ii^	84.32 (11)
O7—Cu1—Cu1^i^	169.25 (15)	O14—Cu2—Cu2^ii^	175.37 (14)
O1—C1—O2	126.3 (5)	O8—C17—O9	125.4 (5)
O1—C1—C2	116.3 (5)	O8—C17—C18	116.9 (4)
O2—C1—C2	117.4 (5)	O9—C17—C18	117.6 (5)
C3—C2—C7	120.4 (5)	C23—C18—C19	121.4 (5)
C3—C2—C1	120.7 (5)	C23—C18—C17	119.4 (5)
C7—C2—C1	118.9 (5)	C19—C18—C17	119.1 (4)
C4—C3—C2	120.0 (6)	C20—C19—C18	118.8 (5)
C4—C3—H3	120.0	C20—C19—H19	120.6
C2—C3—H3	120.0	C18—C19—H19	120.6
C3—C4—C5	120.2 (5)	O10—C20—C19	124.8 (5)
C3—C4—H4	119.9	O10—C20—C21	115.3 (5)
C5—C4—H4	119.9	C19—C20—C21	119.9 (5)
C4—C5—C6	119.9 (5)	C22—C21—C20	120.0 (5)
C4—C5—H5	120.1	C22—C21—H21	120.0
C6—C5—H5	120.1	C20—C21—H21	120.0
O3—C6—C7	124.6 (5)	C21—C22—C23	120.6 (5)
O3—C6—C5	115.5 (5)	C21—C22—H22	119.7
C7—C6—C5	119.9 (5)	C23—C22—H22	119.7
C2—C7—C6	119.4 (5)	C18—C23—C22	119.2 (6)
C2—C7—H7	120.3	C18—C23—H23	120.4
C6—C7—H7	120.3	C22—C23—H23	120.4
O3—C8—H8A	109.5	O10—C24—H24A	109.5
O3—C8—H8B	109.5	O10—C24—H24B	109.5
H8A—C8—H8B	109.5	H24A—C24—H24B	109.5
O3—C8—H8C	109.5	O10—C24—H24C	109.5
H8A—C8—H8C	109.5	H24A—C24—H24C	109.5
H8B—C8—H8C	109.5	H24B—C24—H24C	109.5
O4—C9—O5	125.2 (5)	O12—C25—O11	124.5 (5)
O4—C9—C10	117.3 (5)	O12—C25—C26	117.1 (4)
O5—C9—C10	117.4 (5)	O11—C25—C26	118.4 (4)
C11—C10—C15	119.7 (5)	C27—C26—C31	119.3 (5)
C11—C10—C9	121.5 (5)	C27—C26—C25	119.9 (5)
C15—C10—C9	118.6 (5)	C31—C26—C25	120.7 (4)
C10—C11—C12	119.4 (6)	C28—C27—C26	119.1 (5)
C10—C11—H11	120.3	C28—C27—H27	120.5
C12—C11—H11	120.3	C26—C27—H27	120.5
C13—C12—C11	121.0 (6)	C29—C28—C27	121.3 (5)
C13—C12—H12	119.5	C29—C28—H28	119.3
C11—C12—H12	119.5	C27—C28—H28	119.3
C12—C13—C14	119.5 (6)	C28—C29—C30	119.8 (5)
C12—C13—H13	120.2	C28—C29—H29	120.1
C14—C13—H13	120.2	C30—C29—H29	120.1
C15—C14—O6	124.8 (6)	C31—C30—O13	124.5 (5)
C15—C14—C13	120.4 (6)	C31—C30—C29	119.7 (5)
O6—C14—C13	114.8 (5)	O13—C30—C29	115.8 (5)
C14—C15—C10	119.9 (5)	C30—C31—C26	120.6 (5)
C14—C15—H15	120.0	C30—C31—H31	119.7
C10—C15—H15	120.0	C26—C31—H31	119.7
O6—C16—H16A	109.5	O13—C32—H32A	109.5
O6—C16—H16B	109.5	O13—C32—H32B	109.5
H16A—C16—H16B	109.5	H32A—C32—H32B	109.5
O6—C16—H16C	109.5	O13—C32—H32C	109.5
H16A—C16—H16C	109.5	H32A—C32—H32C	109.5
H16B—C16—H16C	109.5	H32B—C32—H32C	109.5
C1—O1—Cu1	123.6 (3)	C17—O8—Cu2	123.5 (3)
C1—O2—Cu1^i^	120.6 (3)	C17—O9—Cu2^ii^	122.0 (3)
C6—O3—C8	117.0 (5)	C20—O10—C24	119.4 (5)
C9—O4—Cu1^i^	126.7 (3)	C25—O11—Cu2	123.2 (3)
C9—O5—Cu1	118.7 (3)	C25—O12—Cu2^ii^	123.2 (3)
C14—O6—C16	118.1 (5)	C30—O13—C32	117.6 (4)
Cu1—O7—H7A	127 (4)	Cu2—O14—H14B	120 (4)
Cu1—O7—H7B	123 (4)	Cu2—O14—H14A	128 (4)
H7A—O7—H7B	109 (3)	H14B—O14—H14A	108 (3)
			
O1—C1—C2—C3	12.5 (7)	O8—C17—C18—C23	2.6 (7)
O2—C1—C2—C3	−166.2 (5)	O9—C17—C18—C23	−179.6 (5)
O1—C1—C2—C7	−169.6 (5)	O8—C17—C18—C19	−173.4 (5)
O2—C1—C2—C7	11.8 (7)	O9—C17—C18—C19	4.4 (7)
C7—C2—C3—C4	−4.4 (9)	C23—C18—C19—C20	−2.3 (8)
C1—C2—C3—C4	173.6 (5)	C17—C18—C19—C20	173.7 (5)
C2—C3—C4—C5	3.6 (10)	C18—C19—C20—O10	−178.2 (5)
C3—C4—C5—C6	0.0 (10)	C18—C19—C20—C21	1.6 (8)
C4—C5—C6—O3	178.1 (6)	O10—C20—C21—C22	179.4 (5)
C4—C5—C6—C7	−2.9 (10)	C19—C20—C21—C22	−0.4 (9)
C3—C2—C7—C6	1.4 (8)	C20—C21—C22—C23	−0.3 (10)
C1—C2—C7—C6	−176.5 (5)	C19—C18—C23—C22	1.6 (9)
O3—C6—C7—C2	−178.9 (5)	C17—C18—C23—C22	−174.3 (5)
C5—C6—C7—C2	2.2 (9)	C21—C22—C23—C18	−0.3 (10)
O4—C9—C10—C11	−178.6 (5)	O12—C25—C26—C27	−179.6 (5)
O5—C9—C10—C11	3.5 (7)	O11—C25—C26—C27	−2.0 (7)
O4—C9—C10—C15	7.5 (7)	O12—C25—C26—C31	−2.3 (7)
O5—C9—C10—C15	−170.4 (5)	O11—C25—C26—C31	175.4 (5)
C15—C10—C11—C12	−1.2 (8)	C31—C26—C27—C28	−2.8 (8)
C9—C10—C11—C12	−175.1 (5)	C25—C26—C27—C28	174.6 (5)
C10—C11—C12—C13	2.6 (9)	C26—C27—C28—C29	0.5 (10)
C11—C12—C13—C14	−1.7 (10)	C27—C28—C29—C30	2.2 (10)
C12—C13—C14—C15	−0.6 (9)	C28—C29—C30—C31	−2.6 (9)
C12—C13—C14—O6	−179.8 (6)	C28—C29—C30—O13	176.8 (6)
O6—C14—C15—C10	−179.0 (5)	O13—C30—C31—C26	−179.1 (5)
C13—C14—C15—C10	1.9 (8)	C29—C30—C31—C26	0.3 (8)
C11—C10—C15—C14	−1.0 (8)	C27—C26—C31—C30	2.4 (8)
C9—C10—C15—C14	173.0 (5)	C25—C26—C31—C30	−175.0 (5)
O2—C1—O1—Cu1	0.7 (7)	O9—C17—O8—Cu2	−4.5 (7)
C2—C1—O1—Cu1	−177.8 (3)	C18—C17—O8—Cu2	173.2 (3)
O1—C1—O2—Cu1^i^	−3.4 (7)	O8—C17—O9—Cu2^ii^	3.9 (7)
C2—C1—O2—Cu1^i^	175.1 (3)	C18—C17—O9—Cu2^ii^	−173.8 (3)
C7—C6—O3—C8	15.7 (10)	C19—C20—O10—C24	2.3 (8)
C5—C6—O3—C8	−165.3 (6)	C21—C20—O10—C24	−177.5 (6)
O5—C9—O4—Cu1^i^	3.4 (7)	O12—C25—O11—Cu2	0.4 (7)
C10—C9—O4—Cu1^i^	−174.3 (3)	C26—C25—O11—Cu2	−177.1 (3)
O4—C9—O5—Cu1	−3.0 (7)	O11—C25—O12—Cu2^ii^	−1.9 (7)
C10—C9—O5—Cu1	174.7 (3)	C26—C25—O12—Cu2^ii^	175.6 (3)
C15—C14—O6—C16	1.4 (9)	C31—C30—O13—C32	19.3 (9)
C13—C14—O6—C16	−179.5 (6)	C29—C30—O13—C32	−160.1 (5)

Symmetry codes: (i) −*x*+1, −*y*+1, −*z*; (ii) −*x*+2, −*y*+1, −*z*+1.

### Hydrogen-bond geometry (Å, º)

**Table:**

*D*—H···*A*	*D*—H	H···*A*	*D*···*A*	*D*—H···*A*
O7—H7*A*···O3^iii^	0.84 (2)	2.11 (3)	2.912 (6)	161 (5)
O7—H7*B*···O1^iv^	0.83 (2)	2.42 (5)	3.107 (6)	141 (7)
O7—H7*B*···O5^iv^	0.83 (2)	2.60 (5)	3.306 (6)	144 (6)
O14—H14*B*···O13^v^	0.84 (2)	2.00 (2)	2.831 (6)	169 (8)
O14—H14*A*···O11^vi^	0.83 (2)	2.11 (3)	2.905 (5)	160 (6)
O14—H14*A*···O14^vi^	0.83 (2)	2.57 (5)	3.055 (8)	118 (5)
C23—H23···O6^vii^	0.93	2.52	3.355 (7)	149
C27—H27···O6^viii^	0.93	2.57	3.114 (7)	117

Symmetry codes: (iii) *x*, −*y*+3/2, *z*−1/2; (iv) −*x*+1, −*y*+2, −*z*; (v) *x*, −*y*+1/2, *z*+1/2; (vi) −*x*+2, −*y*, −*z*+1; (vii) −*x*+1, *y*−1/2, −*z*+1/2; (viii) *x*+1, −*y*+1/2, *z*+1/2.
